# Sex-dependent effects of early life stress on reinforcement learning and limbic cortico-striatal functional connectivity

**DOI:** 10.1016/j.ynstr.2022.100507

**Published:** 2022-12-05

**Authors:** Katharina Zühlsdorff, Laura López-Cruz, Ethan G. Dutcher, Jolyon A. Jones, Claudia Pama, Stephen Sawiak, Shahid Khan, Amy L. Milton, Trevor W. Robbins, Edward T. Bullmore, Jeffrey W. Dalley

**Affiliations:** a Department of Psychology, University of Cambridge, Downing Site, Cambridge, CB2 3EB, UK; b Behavioural and Clinical Neuroscience Institute, University of Cambridge, CB2 3EB, UK; c Faculty of Science, Technology, Engineering & Mathematics, The Open University, Walton Hall, Kents Hill, Milton Keynes, MK7 6AA, UK; d Department of Psychiatry, Herchel Smith Building for Brain and Mind Sciences, Forvie Site, Cambridge, CB2 0SZ, UK; e Department of Physiology, Development and Neuroscience, University of Cambridge, Downing Site, Cambridge, CB2 3EB, UK; f Wolfson Brain Imaging Centre, Department of Clinical Neurosciences, University of Cambridge, Cambridge Biomedical Campus, Box 65, Cambridge, CB2 0QQ, UK; g GlaxoSmithKline Research & Development, Stevenage, UK

## Abstract

Major depressive disorder (MDD) is a stress-related condition hypothesized to involve aberrant reinforcement learning (RL) with positive and negative stimuli. The present study investigated whether repeated early maternal separation (REMS) stress, a procedure widely recognized to cause depression-like behaviour, affects how subjects learn from positive and negative feedback. The REMS procedure was implemented by separating male and female rats from their dam for 6 h each day from post-natal day 5–19. Control rat offspring were left undisturbed during this period. Rats were tested as adults for behavioral flexibility and feedback sensitivity on a probabilistic reversal learning task. A computational approach based on RL theory was used to derive latent behavioral variables related to reward learning and flexibility. To assess underlying brain substrates, a seed-based functional MRI connectivity analysis was applied both before and after an additional adulthood stressor in control and REMS rats. Female but not male rats exposed to REMS stress showed increased response ‘stickiness’ (repeated responses regardless of reward outcome). Following repeated adulthood stress, reduced functional connectivity from the basolateral amygdala (BLA) to the dorsolateral striatum (DLS), cingulate cortex (Cg), and anterior insula (AI) cortex was observed in females. By contrast, control male rats exposed to the second stressor showed impaired learning from negative feedback (i.e., non-reward) and reduced functional connectivity from the BLA to the DLS and AI compared to maternally separated males. RL in male rats exposed to REMS was unaffected. The fMRI data further revealed that connectivity between the mOFC and other prefrontal cortical and subcortical structures was positively correlated with response ‘stickiness’. These findings reveal differences in how females and males respond to early life adversity and subsequent stress. These effects may be mediated by functional divergence in resting-state connectivity between the basolateral amygdala and fronto-striatal brain regions.

## 1 Introduction

Major depressive disorder (MDD) is primarily characterized as an affective disorder and is one of the leading causes of disability worldwide (APA, 2013). The brain mechanisms that mediate MDD are not yet fully known, and only approximately 50% of people respond to first-line antidepressant medication (Garcia-Toro et al., 2012). Impaired decision-making and cognitive function are also hallmark features of MDD (Cella et al., 2010; Lam et al., 2014; Perini et al., 2019; Wang et al., 2014). Reinforcement learning (RL) is the process by which feedback from the environment, such as reward or punishment, is used to adjust behaviour so as to maximize rewards (Sutton and Barto, 1998). RL is increasingly recognized to be impaired in patients diagnosed with MDD (Admon and Pizzagalli, 2015; Rothkirch et al., 2017; Rupprechter et al., 2018). New insights into the etiology of this disorder may emerge by further research into the mechanisms underlying these disruptions in RL, which may adversely affect decision-making (Dayan and Daw, 2008).

Accumulating evidence indicates that patients with MDD are impaired on tasks that depend on prior reinforcement to update ongoing behaviour, notably reversal learning tasks where individuals must flexibly adjust behaviour in response to sudden shifts in reward contingencies (Chen et al., 2015; Murphy et al., 2003). In recent years, it has been shown that RL models can be fit to reversal learning task data, tracking how an individual's expected value of response outcome changes on a trial-by-trial basis (Daw, 2009). Such models include free parameters to explain the observed data and characterize variables of neurobiological interest. Commonly used parameters include the learning rate, which provides a measure of how quickly the expected value is updated based on feedback; the temperature parameter, which measures whether the animal exploits previous information or explores other options; and the ‘stickiness’ parameter, which represents the tendency to select the same choice regardless of prior outcome (Miller et al., 2019). RL models are becoming increasingly used to study psychiatric disorders and have contributed to establishing translational links between preclinical studies in experimental animals and neuropsychiatric disorders in humans (Clarke et al., 2014; F. Wu et al., 2020). After fitting RL models to performance on a probabilistic reversal task (PRL) task in humans, lower learning rates and lower reinforcement sensitivity in MDD patients have been reported. These findings imply that MDD patients learn more slowly from feedback received on previous trials (Chase et al., 2010; Mukherjee et al., 2020). Such a deficit may be influenced by sex effects given the higher prevalence of depression in females compared with males (Bromet et al., 2011; Salk et al., 2017). Indeed, previous research has shown that females are differentially sensitive to reward and punishment signals on decision-making tasks (Fattore, 2015; van den Bos et al., 2013).

Experimental approaches in rodents have enabled researchers to investigate endophenotypes of translational relevance to complex psychiatric disorders such as anxiety, behavioral flexibility, and anhedonia (Cope et al., 2012; Gottesman and Gould, 2003; Iacono, 2018; Laughlin et al., 2011; Moreno-Fernández et al., 2017). A widely used procedure to investigate depression-like behaviours in rodents is repeated early life maternal separation (REMS), where offspring are intermittently separated from their mothers from an early postnatal age until the start of adolescence (Vetulani, 2013). REMS results in an altered behavioral profile including increased depression-like behaviours and anxiety, increased anhedonia as measured by the sucrose preference test (SPT) and reduced appetitively-conditioned locomotor activity in a novel environment (Cui et al., 2020; LeDoux and Pine, 2016; Matthews et al., 1996). The behavioral changes resulting from ELS also show sex-dependent differences; for example, with females exhibiting depressive-like symptoms on the SPT (Goodwill et al., 2019; Kundakovic et al., 2013). In female mice, rule-reversal learning is impaired as a consequence of early life stress (ELS) (Goodwill et al., 2018). To date, however, it has not been investigated how RL in rodents is affected following ELS.

Resting-state fMRI studies have helped identify regions of the brain with altered connectivity in MDD. Reductions in resting-state functional connectivity (rsFC) from the amygdala to the prefrontal cortex (PFC) are widely reported in medication-naïve patients and people that have experienced ELS (Fan et al., 2014; Herringa et al., 2013; Satterthwaite et al., 2016; M. Wu et al., 2020). Sex-dependent effects on rsFC have also been found in MDD, such as in the caudate nucleus, middle frontal gyrus and posterior cingulate gyrus (Mei et al., 2022; Zheng et al., 2022). Previous research has demonstrated that following 8 weeks of SSRI antidepressant treatment in humans, PFC and amygdala activity could be normalized and was indistinguishable from controls (Fales et al., 2009; Zhang et al., 2020). These findings implicate amygdala-PFC circuitry in MDD etiology and treatment response.

In the present study, we used a seed-based fMRI approach to investigate functional connectivity strength in animals exposed to REMS between post-natal days (PND) 5–19 followed by repeated adulthood shock stressors, to which all animals were exposed. This analysis was combined with RL modelling to fit behavioral variables obtained during a touchscreen based PRL task. The PRL task was used because it includes 'spurious' feedback, with 20% of incorrect trials being rewarded (and 80% of correct trials). Thus, stimulus ambiguity and uncertainty are introduced, to which depressed individuals are less tolerant (Murphy et al., 2003; Saulnier et al., 2019). The extracted parameters were compared with conventional measures of reversal learning performance such as win-stay/lose-shift behaviour and perseverative responding. Lower learning rates for rewarded and non-rewarded trials in the REMS group were predicted, along with a lower sensitivity of choice based on reinforcement history, as measured via the exploitation vs exploration parameter, 'temperature'. Furthermore, we expected that the repeated adulthood stress would have an additive effect, with these parameters being impacted further in REMS animals, and reduced in control animals. If no effects of ELS were observed, we hypothesized that these parameter impairments would be revealed following exposure to an additional stressor later in life. We further predicted that altered learning and feedback sensitivity would be reflected by abnormalities in the functional connectivity of the amygdala with anterior cortical regions. Similarly to behavior, it was expected that both REMS and adulthood stress would additively contribute to alterations in functional connectivity, and that in control animals, the repeated shock stressor would also impact rsFC.

## 2 Methods and materials

### 2.1 Subjects

Fourteen pregnant female Lister-Hooded rats were obtained from Envigo (Blackthorn, UK) and were delivered on gestational day 14/15. The pregnant rats gave birth to litters spontaneously on gestational days 22–24. Within three days of birth, litter sizes were adjusted to be between four and six offspring, with each litter consisting of two females and two males except one, which contained two females and four males. Males and females could be distinguished based on physical features, such as size (females tend to be smaller) and anogenital distance (which is larger in males). To reduce epigenetic and genetic effects, pups were mixed when two litters were born within 24 h of each other, which was the case for ten of the litters. If this did not occur, the litters were not mixed. The pups were then assigned to the control or maternally separated (MS) group (n = 28, 14 females, 14 males and n = 30, 14 females, 16 males, respectively). The day of birth was defined as postnatal day zero (PND0). Food and water were provided *ad libitum* up to PND73-78, at which point behavioral testing commenced. Throughout behavioral training animals were kept to 85% of their free feeding weight. The temperature in the home cages was kept constant at 23 ± 0.2 °C and relative humidity was constant at 60 ± 5%. The animals were kept on a 12-h reversed light/dark cycle (lights off at 09:00). Experiments were conducted on Project License PA9FBFA9F, in accordance with both the UK Animals (Scientific Procedures) Act 1986, amendment regulations 2012 and the EU legislation on the protection of animals used for scientific purposes (Directive, 2010/63/EU) following ethical review by the University of Cambridge Animal Welfare and Ethical Review Body (AWERB) and the GSK Policy on the Care, Welfare and Treatment of Animals.

### 2.2 Repeated early maternal separation (REMS)

Pups that were assigned to the MS group were separated each day from their mothers for 6 h a day between PND5 and PND19, starting around 11 a.m.-12:30 p.m. MS duration of 6 h was selected as it has been shown that this maximally disrupts mother-pup interactions that are important for development and results in negative consequences, with shorter periods not eliciting equivalent stress responses (Pryce and Feldon, 2003). The procedure began at PND5 based on previous studies showing that REMS starting at that period of development results in persistent behavioral and brain effects (Matthews et al., 1996; Matthews and Robbins, 2003). The pups were taken to a separate room during this time and were kept together in a small cage in a ventilated cabinet. Fresh bedding was provided to the pups with some nest material from their home cage. The surface temperature of the bedding was maintained between 30 and 35 °C with a heat pad and warmed air. Control animals were kept in their home cages. After PND20, offspring were maintained in same-sex pairs. A timeline of the study is shown in Fig. 1.

**Fig. 1:**
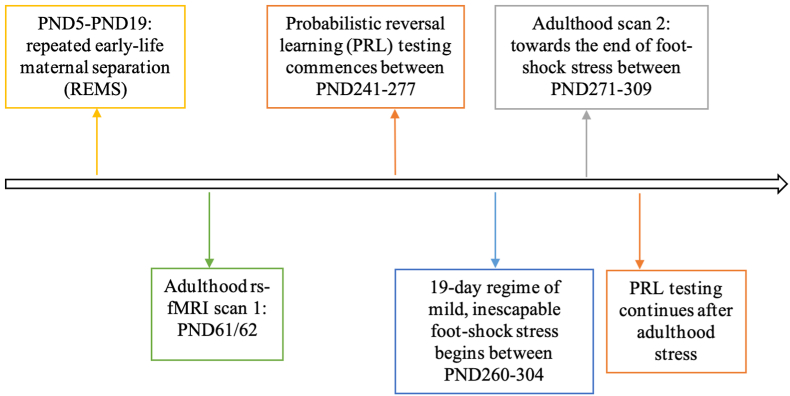
Experimental timeline. Rats were separated from PND5 to 19 prior to behavioral testing on a probabilistic reversal learning (PRL) task and resting-state fMRI (rs-fMRI) before and after exposure to adulthood stress.

### 2.3 Probabilistic reversal learning task

Animals were trained in one of eight identical operant chambers (Med Associates, St. Albans, VT, USA). On one wall, there was a 15-inch LCD touchscreen, and on the opposite side of the chamber, a pellet receptacle with a head-entry detector and accompanying light was located. Small 50% sucrose pellets were delivered by an electronic pellet dispenser (TestDiet, St. Louis, MO, USA) when trials were completed. K-Limbic software (Conclusive Marketing LTD., High Wych, UK) was used to control the touchscreens.

Animals were trained or tested during a single session each day, which automatically ended after 40 min or when 200 trials were completed, whichever came first. After habituation to the test environment, animals were trained to respond to a solid white square stimulus with an ‘X’ in the middle on the right or left side of the touchscreen. If the stimulus was touched, a 0.5 s tone was presented, the pellet receptacle light switched on, and a food pellet was delivered. The stimulus remained illuminated on the screen until the animal touched the screen. When the animal took the reward from the receptacle, the light was turned off and a 5 s inter-trial interval was initiated. After receiving at least 100 rewards during two consecutive sessions, the rats progressed to the next stage, which animals had to respond to the solid white square stimulus, but any touch outside of the stimulus was punished by the house light being on for 5 s, followed by the receptacle light being switched on until the animal made a head entry, after which the receptacle light was turned off and a 5 s ITI began. After 100 rewards on two consecutive sessions, they progressed to the deterministic reversal learning task, during which two white square stimuli on opposite sides of the screen were presented simultaneously. One was the ‘correct’ stimulus and the other one was ‘incorrect’. When the former was touched, the trial was rewarded. If the ‘incorrect’ option was touched, a 5 s punishment was introduced. When the animal selected the ‘correct’ stimulus 8 times consecutively, the contingencies were reversed, and the adjacent side was now the ‘correct’ choice. After animals achieved 4 reversals on two consecutive days, they progressed to the probabilistic reversal learning task. This task was structured similarly to the deterministic reversal learning task, except that in PRL only 80% of trials were rewarded for a ‘correct’ response, and 20% of ‘incorrect’ responses were rewarded randomly. Contingencies were reversed when the animal reached the reversal criterion, which was defined as 8 consecutive responses being correct. A summary of the different stages of the task with figures can be found in the supplementary materials.

PRL testing started at PND241-277 and ended between PND257-290. After adulthood stress (see below), PRL testing was repeated over 3 days (Fig. 1). Since there were only 3 sessions after adulthood stress, the results prior to the stressor were constrained to the first 3 sessions. For each session, trials up until the fourth reversal were included. Forty-nine animals were included in the final analysis, as some animals had to be excluded due to data loss or the animal not completing the task correctly, e.g., by not engaging and not selecting either stimulus throughout the session (control females: 3; MS females: 5; MS males: 1).

### 2.4 MRI image acquisition

Animals were scanned on PND61-62 and between PND271-309 (during exposure to the second stressor) using a 9.4 T horizontal bore MRI scanner (BioSpec 94/20, Bruker, Coventry, UK). Structural imaging was achieved using multi-parametric mapping, from which T1-weighted and PD-weighted sequences were obtained. Contrast between grey and white matter was enhanced by applying magnetization transfer (MT) pulses (10 μT, 2 kHz off-resonance) within each repetition. The field of view of 30.72 × 25.6 × 20.48 mm^3^ was constrained within a matrix of 192 × 160 × 128 voxels, yielding an in-plane isotropic resolution of 0.16 mm^2^. To acquire the functional MRI data, a three-dimensional multi-echo gradient echo sequence was used with a relaxation time (TR) of 1832 ms, an echo time (TE) of 5.5/16.5/27 ms for the three echoes, a flip angle of 6°, and RF spoiling of 117°. The field of view of 28.8 × 21.6 × 21.6 mm^3^ was constrained within a matrix of 64 × 48 × 48 voxels, resulting in an in-plane resolution of 0.45 mm with a slice thickness of 0.5 mm 655 frames were acquired for the resting-state fMRI scans acquired contiguously and interleaved acquisition. An acceleration factor of 1.55 was used.

Rats were kept under 1–2% isoflurane anesthesia which was delivered in 100% oxygen at 1 L/min throughout the duration of the structural sequences, which was lowered to 20% oxygen/80% air during resting-state fMRI acquisition. The animals were continuously monitored using a pulse oximeter, respiratory tracer pad, and rectal temperature probe (SA Instruments, Stony Brook, NY, USA) to ensure that vital signs were within normal limits. Whenever the signs exceeded the limits, anesthesia depth and temperature (using a heat pad) were adjusted. If the vital signs didn't improve, the imaging session was terminated.

### 2.5 Adulthood stress

Both groups of animals were subjected to a regime of mild, inescapable foot-shock stress over a continuous 19-day period, which began between PND260-304. On 14 to 16 of those days, animals were placed in a conventional operant chamber at approximately the same time of day (Med Associates, St Albans, VT, USA) for 30 min during which time they received 1 or 2 temporally unpredictable foot-shocks. The chambers were equipped with a grid floor (connective to a scrambled shock generator (Med Associates, St Albans, VT, USA)), house light, high-contrast distinctive wallpaper and a small overhead camera. Moreover, a distinctive scent cue was added through the application of eucalyptus globulus essential oil (3 drops, Neal's Yard Ltd, Cambridge, UK) to a cotton ball, which was placed in the sound-attenuating box that enclosed each chamber prior to the session. This was done so that the shock environment differed from the PRL testing environment. Every animal received 20 mild shocks in the 19-day time period. During the first 8 days, the number of shocks delivered was 2, 1, 2, 0, 1, 2, 0, 1. On the remaining days, the distribution of shock delivery of the remaining 11 shocks varied between animals. However, the number of shocks in one session would never exceed 2. The timing onset of each shock was random but did not occur during the first five or last 10 min of the sessions. Shock intensity was 0.5 mA with a duration of 0.5 s.

### 2.6 Q-learning models

Three Q-learning models were fit to the probabilistic reversal learning data on a trial-by-trial basis (Daw, 2009) according to the following equation:

(1) $$Q_{t}\left(c_{t}\right)=Q_{t}\left(c_{t}\right)+\alpha \times \left(r-Q_{t}\left(c_{t}\right)\right)$$

where Q_t+1_(c_t_) is the expected value of the lever that was chosen on the next trial, Q_t_(c_t_) is the expected value of the choice taken on the current trial (c_t_), α is the learning rate and r is the reward on the current trial. r − Q_t_(c_t_) is also known as the prediction error. The α parameter influences how much the animal updates the Q-value based on the prediction error, with high α assigning high importance to it in the update function, and low α assigning low importance.

The probability of choosing a lever given the Q-values for each was calculated using the softmax decision rule:

(2) $$P\left(c_{t}=L|Q_{t}\left(L\right),Q_{t}\left(R\right)\right)=\frac{e^{(Q_{t}(L)/\;\beta +\kappa \times L_{t-1}}}{e^{(Q_{t}(L)/\;\beta +\kappa \times L_{t-1}}+e^{(Q_{t}(R)/\;\beta +\kappa \times R_{t-1}}}$$

where Q_t_ (L) and Q_t_ (R) are the Q-values of the left and right levers, respectively, and β is the temperature parameter, which can be interpreted as whether the animal exploits the information it has gained on the previous trial (low β), or whether it explores the other options (high β). The α parameter was set to take values between 0.001 and 1, whereas the β parameter could take values between 0.005 and 5 (Daw, 2009). κ is the autocorrelation parameter, also known as ‘stickiness’ and represents the tendency to select the same choice regardless of reinforcement outcome of a given stimulus on previous trials. The step sizes for the α parameter were set to 0.05, whereas for β they were set to 0.15. L_t−1_ and R_t−1_ indicate whether the left or right lever was selected on the previous trial and take values of 1 or 0, depending on whether that side was chosen or not. κ could take values between −1 (decreased repetition or ‘stickiness’) and 1 (increased repetition or 'stickiness') and had a step size 0.1. A grid-search algorithm tested all the possible parameter combinations for each animal and each session in order to determine the optimal combination for the given set of trials in a session. The step size determines which values within the constrained parameter space were used in the grid search.

Next, the probability of observing the sequence of actions as in the reversal learning task was calculated by the product of probabilities:

(3) $$P\left(Data\;D\;|\;Model\;M\;,parameters\;\theta \right)=\Pi \;P\left(c_{t}=L|Q_{t}\left(L\right),Q_{t}\left(R\right)\right)$$

The free parameters were fit to achieve the maximum likelihood of the probability density function of data D:

(4) argmaxP(D|M,θ)

The three models were based on the one described above, however two of the models included slight modifications. One of the models only had an α and β term, but no κ parameter. The third model included two separate learning terms for α, one for rewarded trials (α_rew_) and one for non-rewarded trials (α_non−rew_). The Q-values in those models are also updated as seen in equation (1). These models were implemented in MATLAB R2019b (Mathworks). The scripts were based on the code for a two-choice reversal learning task (Zhukovsky et al., 2019).

The models were compared using three different measures:

1. Log-likelihood Ratio (d)

$$d=2\left[\log\;P\;\left(D\;|\;M_{2},\theta_{M2}\right)-\log\;P\;\left(D\;|\;M_{1},\theta_{M1}\right)\right]$$

where M1 and M2 are the two models being compared, D is the sequence of data points, and θ are the best fit parameters of that model.

2. Pseudo r^2^

$$pseudo\;r^{2}=\frac{logP\left(D|M,\theta_{M}\right)\;–\;0.5^{n}}{0.5^{n}}$$

Here, n represents the number of trials, D is the sequence of data points, M is the model and θ are the best fit parameters of that model. Pseudo r^2^ is representative of the probability of observing the data given the best fit parameters of that model compared with the probability of observing the data by chance.

3. Bayesian Information Criterion

$$BIC=\log(P(D\left|M))\approx \log(P(D\right|M,\theta_{M})\;–\;(\frac{n}{2})\log(m)$$

n is the number of free parameters, and m is the number of observations. The BIC penalizes the number of parameters, and thus overfitting. Based on these measures, the best models were selected for further analysis.

### 2.7 Statistical analyses of behavioral data

To compare the Q-learning parameters with conventional PRL measures, we also calculated win-stay and lose-shift behaviour after a correct and an incorrect trial (lose-shift correct and lose-shift incorrect, respectively), the number of trials required to reach a reversal (otherwise known as trials to criterion), the proportion of correct responses and number of perseverative responses, which were measured by the number of times the animal would select the same stimulus after a contingency reversal and before pressing the correct stimulus (Jentsch et al., 2002). Win-stay behaviour was calculated as the percentage of trials during which the animal selected the same stimulus as on the previous trial if the previous trial was rewarded. Lose-shift, on the other hand, was the percentage of trials during which the animal shifted its response to the other stimulus after not receiving a reward on the last trial. Data are publicly available at: https://doi.org/10.17863/CAM.90390.

The data were centered, and linear mixed-effects (LME) models were fit to the RL parameters (α_rew_, α_non−rew_, β and κ) and conventional parameters, with MS, sex, adulthood stress and their interaction used as fixed factors, with a random intercept for each subject and subsequent *post-hoc* pairwise comparisons of estimated marginal means (Lenth, 2018; Pinheiro et al., 2021). The residuals were checked for normality using the Shapiro-Wilk test, which was verified in our data and confirmed an assumption required for fitting a LME model (Schielzeth et al., 2020). The relationship between the RL and conventional PRL measures was investigated through a correlation analysis using Pearson's correlation coefficient (PCC), accounting for multiple comparisons with FDR correction of 5% (Benjamini and Hochberg, 1995). Beforehand, it was ensured that all measures followed a normal distribution using the Shapiro-Wilk test, a necessary condition when calculating the PCC. All statistical analyses were run in R (R: A Language and Environment for Statistical Computing (R Core Team, 2020)). For all analyses, significance was at p = 0.05.

### 2.8 Image pre-processing

Thirty-one animals were scanned at PND61/62 (control females: N = 8, MS females: N = 6, control males: N = 8, MS males: N = 9); forty-four animals were scanned at the second time point (control females: N = 10, MS females: N = 7, control males: N = 13, MS males: N = 14). All except four animals that were scanned at the first time point were also scanned at the second time point. The reason for this and only thirty-one animals being scanned at the first time point was due to the scan having to be terminated prematurely because of concerns over the animal's wellbeing (e.g., low respiratory rate) or scanner availability. MT structural scans were pre-processed using SPMmouse (Sawiak et al., 2013) using the Statistical Parametric Mapping software (SPM8) (Friston et al., 1994). The scans were bias-corrected and rigidly registered with the same orientation before a study-specific template was generated using Diffeomorphic Anatomical Registration using Exponential Lie algebra (DARTEL) (Ashburner, 2007).

Resting-state fMRI (rs-fMRI) images were pre-processed using the FMRIB Software Library (FSL) (Smith et al., 2004) and the Analysis of Functional NeuroImages software (AFNI) (Cox, 1996). Every three volumes were averaged as the rs-fMRI images were acquired using a multi-echo sequence and were subsequently bias-corrected in FSL (fast). Next, the functional scans were slice-timing corrected (slicetimer) and despiked (3ddespike). The first 5 vol were discarded to only include steady-state volumes in the EPI sequence, and the scans then underwent motion correction (3dvolreg). Motion outliers were identified (fsl_motion_outliers) using *dvars* as a metric. The volumes in which motion exceeded the box-plot cut-off were censored, however, this was only the case for 1% of total volumes (Smith et al., 2004). The voxel sizes of functional scans were then multiplied by 10 to be comparable to human voxel dimensions, which is required to use FSL registration tools (Bajic et al., 2017).

Functional scans were co-registered to structural scans using 6 DOF affine registration (FLIRT) (Jenkinson and Smith, 2001). MT structural scans were non-linearly registered to the study-specific template (FNIRT) (Smith et al., 2004). Using the warp fields computed by FNIRT, the functional scans were warped into common template space, after applying the pre-transform using the affine matrix from FLIRT (applywarp) (Smith et al., 2004). The final images were high-pass filtered (100s) and smoothed using a FHWM of 1.7 mm. In the supplementary material (supplementary figure (SF). 1), figures of the quality checks can be found, including the estimated mean displacement and a visualization of registration efficacy.

### 2.9 Seed-based fMRI analysis

Region-of-interest (ROI) masks were based on a published atlas (Valdés-Hernández et al., 2011) and warped into the study-specific template space using warp fields generated by DARTEL. The following ROIs were used: prelimbic cortex (PrL), infralimbic cortex (IL), basolateral amygdala (BLA), medial orbitofrontal cortex (mOFC) and lateral orbitofrontal cortex (lOFC). The masks can be found in the supplementary material (SF. 2). The seed-based analysis was run in FSL (dual_regression) (Beckmann et al., 2009). The resulting voxelwise connectivity maps were compared using randomize with 5000 permutations and threshold-free cluster enhancement (Winkler et al., 2014). Multiple comparisons were corrected for using the family-wise error rate. The contrast tested was a sex × MS interaction. Furthermore, the Q-learning parameters α_non-rew_ and κ from the RL model were centered and were separately used as regressors in the BLA and mOFC analyses (which was based on the results of the first seed-based analyses), accounting for sex and REMS. Contrasts tested whether there was a positive or negative correlation with these parameters. FSLeyes was used for data visualization with the right and left hemispheres of the presented fMRI images inverted with respect to the observer's perspective (Smith et al., 2004).

## 3 Results

### 3.1 Behavioral effects of REMS stress on probabilistic reversal learning

An analysis of conventional variables on the PRL task revealed no significant differences in the proportion of correct responses or the number of trials required to successfully complete a reversal in male or female REMS animals compared with controls (SF.5). However, the proportion of trials where an animal selected the same response after receiving a reward (i.e., ‘win-stay’ behaviour), regardless of whether the previous response was correct or incorrect, was significantly lower in females than in males (correct responses: F(1,44) = 8.85, p = 0.0048, η^2^p = 0.17; incorrect responses: F(1,44) = 4.11, p = 0.049, η^2^p = 0.09). Moreover, win-stay after an incorrect response was affected by adulthood stress (F(1,236) = 6.04, p = 0.015, η^2^p = 0.02), with this measure increasing in control females (t(236) = -2.72, p = 0.0071).

A different profile of effects was observed for lose-shift behaviour after correct trials, where a significant sex × MS × adulthood stress interaction was observed (F(1,236) = 4.16, p = 0.043, η^2^p = 0.02) (Fig. 2a). Following a second stressor, lose-shift behaviour after correct trials significantly increased in control males, but not in MS males (t(236) = -2.56, p = 0.011). This dissociation was not evident for female rats. A significant sex × MS × adulthood stress interaction was also observed in lose-shift behaviour following incorrect responses (F(1,236) = 7.49, p = 0.0067, η^2^p = 0.03). Prior to the second stress, female MS rats showed significantly lower lose-shift behaviour compared with female control animals (t(44) = 3.06, p = 0.0040). However, following a second stressor, lose-shift behaviour significantly increased in female MS rats, unlike control females (t(236) = -2.02, p = 0.044) (Fig. 2b). This outcome was not present in males, again demonstrating sexual dimorphism in how male and female rats adapt to repeated stress exposure.

**Fig. 2:**
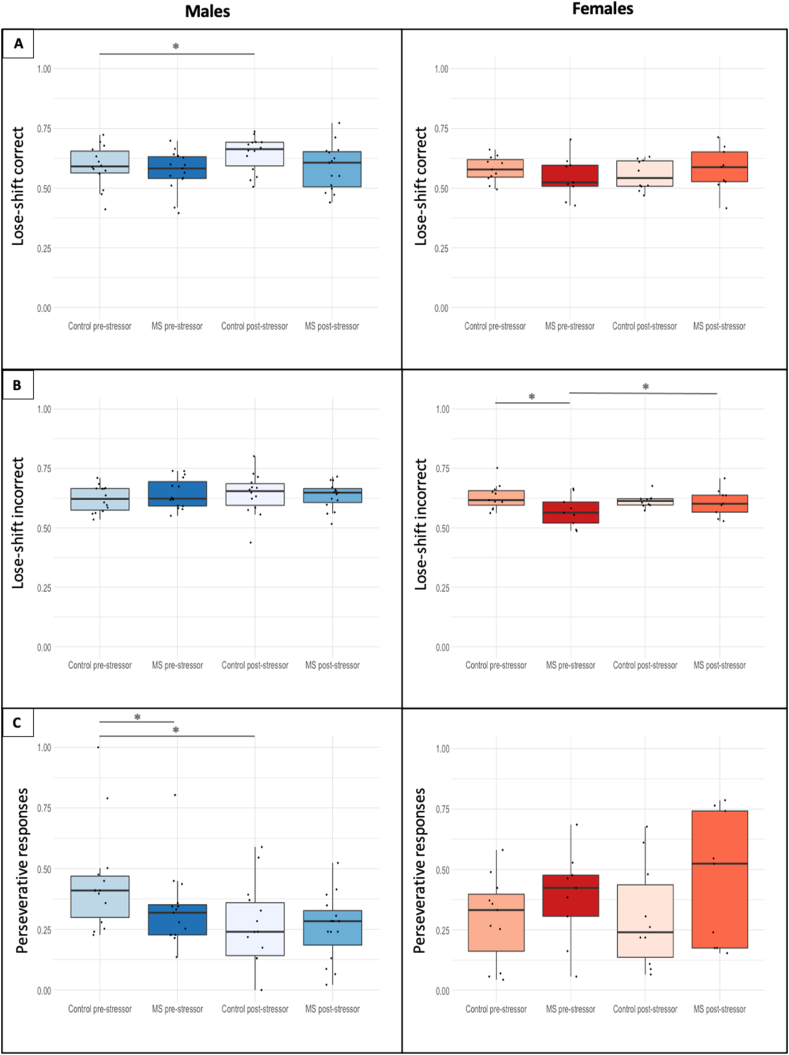
Effects of maternal separation (MS) stress on probabilistic reversal learning task in male (blue) and female (orange) rats before and after a second stress during adulthood. The main variables affected by MS and adulthood stress were perseverative responding and lose-shift behaviour. Following a second stress during adulthood, lose-shift behaviour after a correct trial increased in control males (p = 0.011) but was unchanged in MS males (p = 0.91). MS females shifted less after a loss and an incorrect trial than control females (p = 0.0040), an effect that was increased as a result of adulthood stress in MS females only (p = 0.044). MS males showed lower perseverative responses than control males prior to the adulthood stress (p = 0.0097). Control males only showed a decrease in perseverative responding after the foot-shock stressor (p = 0.010). * – p < 0.05; ** – p < 0.10. (For interpretation of the references to color in this figure legend, the reader is referred to the Web version of this article.)

Our analysis further revealed a sex × adulthood stress interaction (F(1,236) = 8.29, p = 0.0043, η^2^p = 0.03) and a sex × MS interaction (F(1,44) = 7.19, p = 0.010, η^2^p = 0.14) with respect to perseverative responses after a reversal (i.e., repeat responses directed at the incorrect stimulus following a reversal). *Post-hoc* comparisons revealed that MS males made fewer perseverative responses than control males prior to the second stress during adulthood (t(44) = 2.71, p = 0.0097), as well as a decrease in this measure following adulthood stress in control males only (t(44) = 2.69, p = 0.010). Information on body weight and sucrose preference can be found in (Dutcher et al., 2022).

### 3.2 Effects of maternal separation and adulthood stress on Q-learning parameters

Based on our model comparisons, the 4-parameter model including two separate learning rate terms (α) was selected for further analysis (Fig. 3). A more detailed explanation of how the model was chosen can be found in the supplementary materials (SF.1). Analysis of α_non-rew_, the learning rate for non-rewarded trials, revealed a MS × adulthood stress interaction (F(1,236) = 4.14, p = 0.043, η^2^p = 0.02). This interaction was driven by a decreased learning rate on non-rewarded trials (α_non-rew_) in control males after adulthood stress (t(236) = 2.33, p = 0.021) (Fig. 3b), which was not present in MS males (p = 0.41). By contrast, we found no significant effects on α_non-rew_ in female rats either before or after a second stress during adulthood. There were also no significant effects of the REMS procedure or second stress on the α_rew_ and β parameters (Fig. 3c).

**Fig. 3:**
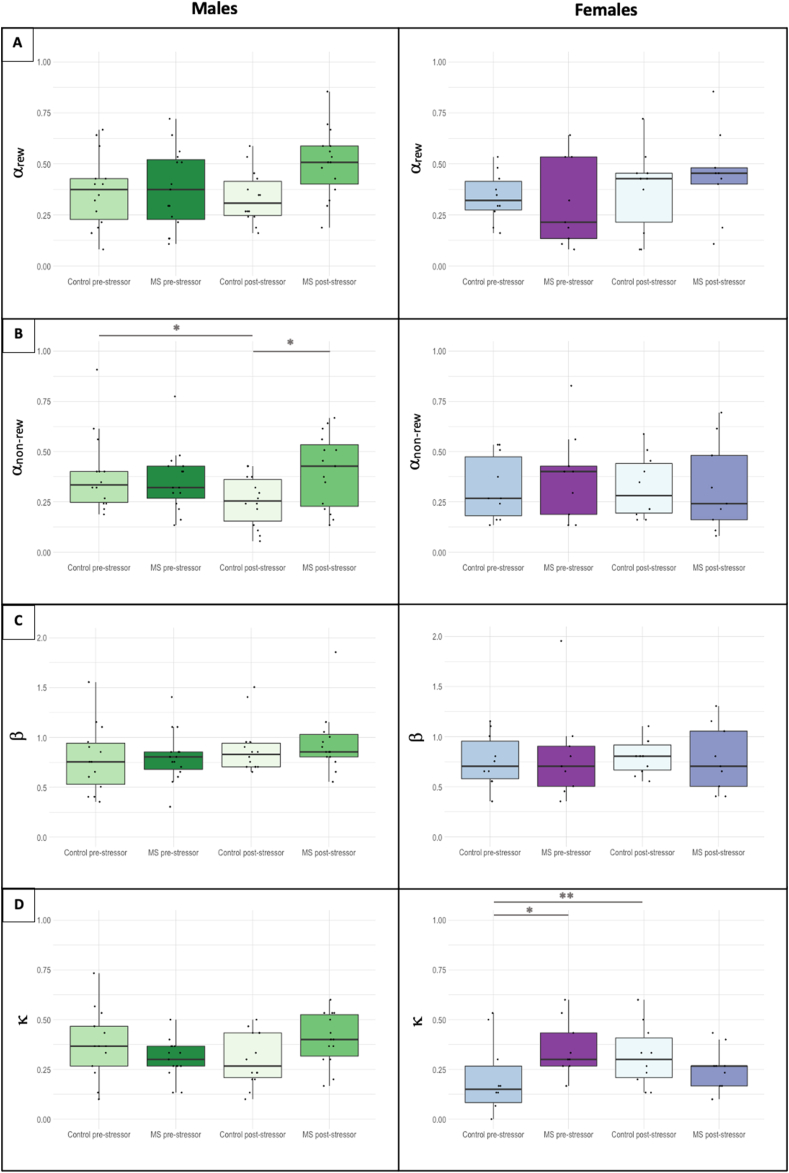
Effect of maternal separation (MS) stress on reinforcement learning parameters on the PRL task in male (green) and female (purple) rats prior to and after a second stress during adulthood. α_rew_ is the learning rate from rewarded trials, α_non-rew_ is the learning rate from non-rewarded trials, ß is the temperature parameter, and κ is the autocorrelation (also known as ‘stickiness’). Repeat stress in adulthood caused a decrease in α_non-rew_ in control males (p = 0.021) but remained unchanged in MS males (p = 0.41). κ increased in control females after the foot-shock stressor (p = 0.055) but not MS females (p = 0.12). * – p < 0.05; ** – p < 0.10. (For interpretation of the references to color in this figure legend, the reader is referred to the Web version of this article.)

LME analysis of κ (“stickiness”) showed a significant sex × MS× adulthood stress interaction (F(1,236) = 8.20, p = 0.0046, η^2^p = 0.03). *Post-hoc* comparisons revealed no significant differences in κ prior to adulthood stress in males but revealed higher values in MS females compared with controls (t(44) = -2.23, p = 0.031) (Fig. 3d). In control females, stickiness increased as a result of the second stressor (t(236) = -1.93, p = 0.055), whereas in MS females there was no difference (p = 0.12) (Fig. 3d). No significant changes in κ were observed in male rats.

### 3.3 Relationship between traditional conventional PRL variables and RL parameters

Correlations between behavioral measures were assessed using Pearson's correlation coefficient. As shown in Fig. 4, we found a positive correlation between κ and win-stay behaviour (r(46) = 0.51, p = 0.002). Thus, despite κ reflecting the same response regardless of outcome, higher values of κ on the PRL task predict increased repeat responses to rewarded stimuli. In addition, there was a positive correlation between learning rate α_rew_ with win-stay correct (r(46) = 0.52, p = 0.002) and the proportion of correct responses (r(46) = 0.56, p = 0.001). Therefore, the faster the animal learns to adjust its behaviour based on reward, the higher its tendency for win-stay behaviour. No other correlations between RL and conventional behavioral measures reached statistical significance.

**Fig. 4:**
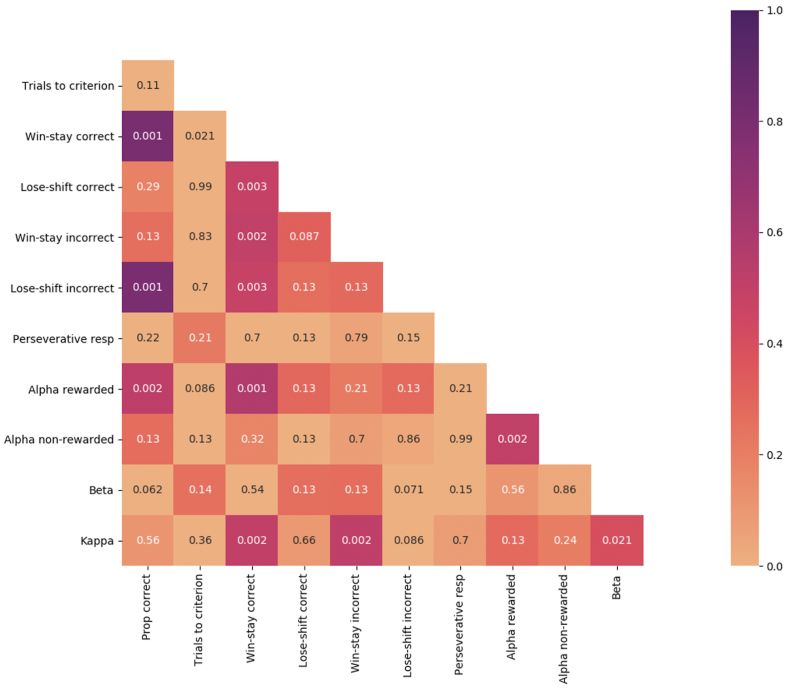
Correlation matrix showing the relationship between conventional probabilistic reversal learning (PRL) measures and parameters from the reinforcement learning model across all sessions of the PRL task. The color bar on the right-hand side represents the correlation strength, the numbers within each square are p-values. Prop correct – proportion of correct responses; Perseverative resp – number of perseverative responses after a reversal; Alpha rewarded – α_rew_ parameter representing the learning rate from rewarded trials; Alpha non-rewarded – α_non-rew_ parameter representing the learning rate from non-rewarded trials; Beta – the exploitation/exploration parameter β; Kappa – κ, also known as the ‘stickiness’ parameter; correct and incorrect indicate the outcome of the previous response. (For interpretation of the references to color in this figure legend, the reader is referred to the Web version of this article.)

In summary, female MS rats showed increased response stickiness on the PRL task prior to a second stress exposure during adulthood. This was accompanied by reduced lose-shift behaviour following an incorrect trial. However, following the second stressor in adulthood, control female animals showed an increase in stickiness, whereas in MS females, lose-shift behaviour after a correct trial increased. In contrast, REMS only affected perseverative responding in males, with MS males perseverating less. Following a second stress, lose-shift behaviour after a correct response increased in control male rats, which corresponded to a significant decrease in the learning rate for non-rewarded trials (i.e., α_non-rew_).

### 3.4 Sexually dimorphic effects of maternal separation stress on functional brain connectivity

A seed-based analysis revealed specific abnormalities in rsFC following REMS and repeated foot-shock stress during adulthood. At PND61/62 (i.e., prior to adulthood stress), no significant alterations in rsFC were observed. However, following an additional stressor, significant rsFC differences were found when the BLA was chosen as the seed region. Details on the extent of the significant clusters and their respective statistics are shown in Fig. 5. Connectivity from the BLA to regions including the anterior insula (AI), cingulate cortex (Cg), IL and dorsolateral striatum (DLS) was affected bilaterally. Specifically, connectivity from the BLA to these regions was decreased in MS females, but increased in MS males, compared with their respective controls. Separate t-tests confirmed that in females, connectivity to all mentioned regions was affected, but in males only connectivity to the DLS and AI was significantly altered.

**Fig. 5:**
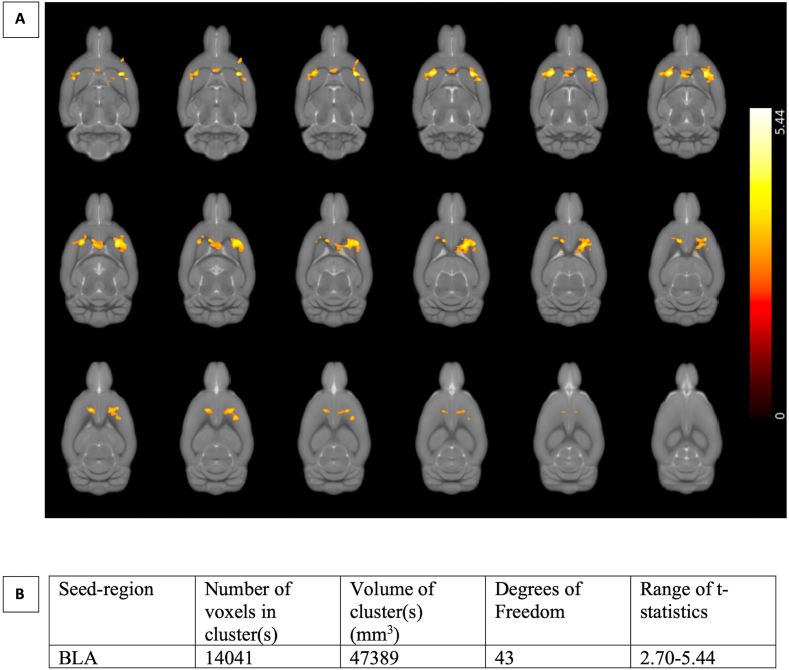
A) Brain regions where resting-state functional connectivity from the basolateral amygdala (BLA) contributes to a significant sex × maternal separation interaction. These include the cingulate cortex, infralimbic cortex, dorsal striatum, and anterior insular cortex. The results show that the connectivity strength is higher in control females and MS males than MS females and control males. The color bar on the right-hand side represents the t-statistic of the respective voxels. B) Table summarizing the main characteristics of the highlighted clusters. (For interpretation of the references to color in this figure legend, the reader is referred to the Web version of this article.)

Furthermore, when voxel-wise connectivity maps of the mOFC were compared between groups, it was found that MS females showed reduced functional connectivity between the mOFC and a cluster located in the ventral striatum (VS) (SF. 4). MS males, by contrast, showed increased functional connectivity between these regions compared with male controls. When the IL, PrL and lOFC were used as seed regions, no significant sex × MS interactions were observed. Based on this evidence, it is likely that the observed differences in rsFC was caused by exposing MS rats to a second bout of unpredictable and uncontrollable stress.

### 3.5 Neural substrates of κ

**Fig. 6:**
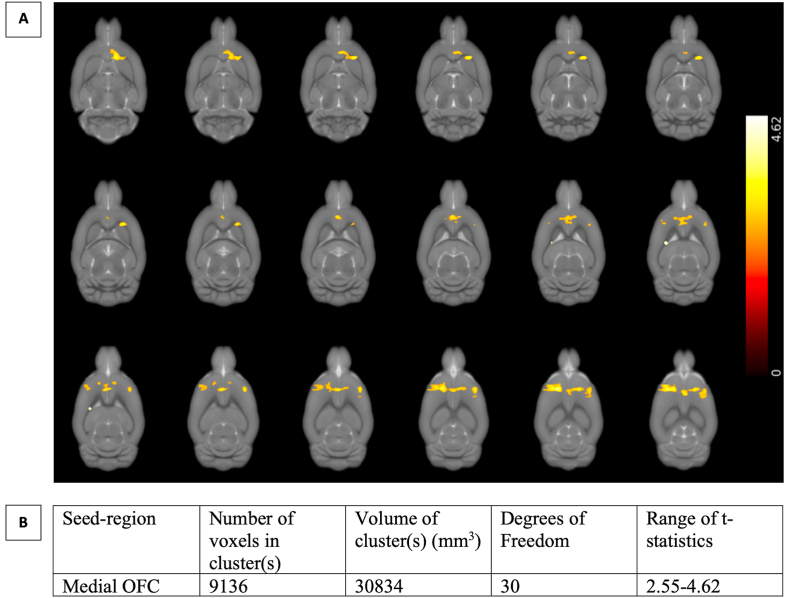
A) Brain regions where resting state connectivity to the medial orbitofrontal cortex (mOFC) is positively correlated with the reinforcement learning parameter κ. The significant cluster spans the cingulate cortex, infralimbic/prelimbic cortices, dorsal striatum, and parts of the granular/dysgranular insular cortex. This effect is seen bilaterally but is more pronounced in the left hemisphere. The color bar on the right-hand side represents the p-values of the respective voxels. B) Table summarizing the main characteristics of the highlighted clusters. (For interpretation of the references to color in this figure legend, the reader is referred to the Web version of this article.)

At PND61/62, κ values for all animals correlated positively with connectivity between the mOFC and a cluster spanning the Cg, dorsal striatum (DS), IL, PrL and the granular/dysgranular insular cortex (Fig. 6). Thus, greater connectivity between the mOFC and these areas predicts higher average values of the RL stickiness parameter κ. No other significant correlations were found.

In summary, we report no significant differences in rsFC caused solely by the REMS procedure. Significant effects in rsFC were only evident following a second uncontrollable stressor with functionally opposed changes in connectivity strength between the BLA and cortico-striatal areas in male and female MS rats. These findings support the notion that early life adversity sets the conditions for stress impacts later in life that are shaped by sex-dependent processes.

## 4 Discussion

### 4.1 Behavioral findings

This study investigated how sex, REMS and adulthood stress interact to determine how subjects learn from positive and negative feedback on a PRL task. The study benefitted from the inclusion of females and males, as well as the availability of behavioral and rs-fMRI data. This made it possible to gain a unique insight into the neural correlates of cognitive flexibility and RL and identify how they differ between sexes. By fitting RL models to the PRL data, we were able to reveal the latent mechanisms underlying behaviour on this task. We observed sexual dimorphic effects of stress on response stickiness (κ) and the learning rate from non-rewarded trials (α_non-rew_). Specifically, we found that females subjected to MS stress show higher κ values than controls, consistent with greater stimulus-response habitual responding. However, the κ parameter increased in control females after the second stressor but did not change in MS females. Therefore, the two forms of early and late stress increased the tendency of females to respond inflexibly but did not interact additively, which was contrary to our expectations. This conclusion is supported by the finding that MS females shifted less after a loss following an incorrect response (i.e., lose-shift incorrect) prior to adulthood stress. As κ is a measure of stickiness, these findings suggest that higher values of κ lead to reduced contingency shifting after a loss in females exposed to stress. In males, by contrast, the conventional perseverative responding measure was significantly lower in the MS group. This measure also decreased following adulthood stress in control males only and was accompanied by a reduction in α_non-rew_ and an increase in lose-shift behaviour. Thus, behaviour under uncertainty was affected in a contrasting, almost mirror-like manner by short- and longer-term stressors in females and males: with increasing tendencies to perseverate in females and increasing tendencies to shift in males.

### 4.2 Connectivity findings

The seed-based neuroimaging results similarly showed opposite changes in females and males. Prior to being exposed to repeated, inescapable foot-shock stress, there were no observable differences in rsFC. However, following stress exposure, significant differences in connectivity were found. Specifically, MS females and males exhibited lower and higher connectivity strength, respectively, between the BLA seed and the IL, Cg, AI and the DLS compared with sex-matched controls. We also report a positive correlation between κ and the connectivity from the mOFC to similar regions, including the IL, DLS and Cg, independent of REMS or sex.

### 4.3 Impact of ELS on habitual behavior and cognitive flexibility

To our knowledge, the stickiness parameter κ, which represents the tendency to select the same choice regardless of prior outcomes, has not previously been explored in the context of depression in humans or REMS in rodents. Habitual behaviour results from the direct elicitation of the instrumental response to the environmental stimuli repeatedly associated with it, autonomous of the goal (Robbins and Costa, 2017). The parameter κ is a putative measure of habitual behaviour manifesting itself in the form of value-free perseverative responding, which is supported by the fact that both behaviours are defined by performing the same response irrespective of whether the action leads to a reward or not (Ito and Doya, 2009; Kanen et al., 2019; Miller et al., 2019).

ELS has been associated with enhanced habitual responding in an avoidance learning paradigm in humans and stress is widely known to bias actions toward habit-based rather than goal-directed control in both rats and humans (Dias-Ferreira et al., 2009; Gordon et al., 2020; Patterson et al., 2019; Schwabe and Wolf, 2009, 2010). Both REMS and chronic adolescent stress in rodents have resulted in impaired performance on cognitive flexibility paradigms (Hyer et al., 2021; Thomas et al., 2016). This has been particularly apparent on PRL tasks, with uncontrollable foot-shock stress in adolescence resulting in increased perseverative responding on this task, an effect not observed following controllable stress (Goodwill et al., 2018; Sanchís-Ollé et al., 2019). In humans, both cognitive flexibility and instrumental learning have been shown to be negatively affected following ELS (Harms et al., 2018). These findings align with the results reported in females, with REMS as well as adulthood stress both increasing κ and impacting performance on the PRL task. However, response stickiness remained unchanged in male subjects and no significant effects of ELS on PRL performance were observed in this group.

### 4.4 ELS differentially impacts females and males

Sex-dependent effects of stress are widely reported in rodents, including differences in anticipatory responding to reward, anxiety-related behaviours and emotionality (Couto et al., 2012; Goodwill et al., 2019; Renard et al., 2007; Vetulani, 2013). There is no consensus on which sex displays greater anxious and depressive phenotypes as a result of ELS in rodents. In humans, on the other hand, MDD is known to be more prevalent in females and women that have experienced childhood trauma tend to be more susceptible to disorders of anxiety and depression, aligning with the observations reported in this study (Albert, 2015; Gallo et al., 2018).

### 4.5 Resilience following REMS

The finding of a significant effect of stress on lose-shift behaviour and α_non-rew_ after adulthood stress in control males, but not MS males, was contrary to our hypotheses and findings from human studies, which suggest that depressed individuals learn more slowly from previous feedback (Mukherjee et al., 2020; Safra et al., 2019). The α_rew_ and β parameters were unaffected by either stressor, which was also unexpected, as both parameters were hypothesized to decrease following stress. Even though REMS has been shown to produce anxiety and depression-like phenotypes in adult rats, several studies have reported no effect of this intervention on depressive-like behaviour (Cui et al., 2006; Raineki et al., 2012; Rice et al., 2008; van der Kooij et al., 2015). Some studies have even demonstrated an improvement in reversal learning performance after MS (Wang et al., 2015). Thus, there is some inconsistency in the behavioral profile caused by REMS, with effects that may be quite subtle. A recent meta-analysis suggested that the discrepancies may be attributable to differences in experimental procedures, such as duration of separation and rodent strain used (Ou-Yang et al., 2022). Maternal behavior is also an important factor, as dams that show increased maternal care post-separation have been shown to attenuate the effects of the separation (Millstein and Holmes, 2007). However, the maternal behavior of dams was not measured in this study, a factor which may have had a differential impact on males and females.

Nevertheless, this unexpected finding is possibly consistent with increased stress resilience in MS males, but not females, agreeing with the concept of stress immunization (Levine, 1957). It may also align with the match/mismatch hypothesis, which suggests that early life experiences support the development of strategies that can be employed in similar situations later in life (Santarelli et al., 2017; Schmidt, 2011). In other words, ELS that is relatively mild in the form of REMS may build the resilience of males to subsequent stress responses. It has been suggested that resilience to adulthood stress may arise due to the MS procedure taking place at the same time every day and thus being predictable (Shi et al., 2021). In this study, pups underwent the procedure at approximately the same time, providing a possible explanation for the apparent resilience to later life stress in males. The reduction of α_non-rew_ in control males may more closely reflect previous reports, since they did not experience ELS and thus were more susceptible to the effects of adulthood stress, resulting in a depression-like phenotype.

### 4.6 Discrepancies between PRL results and other depression-related measures

Further results arising from this study, reported separately, suggest that there were no effects of ELS or adulthood stress on other measures commonly used to investigate depression-like phenotypes, such as sucrose preference and body weight (Dutcher et al., 2022). Although this does not align with the PRL and RL results presented here, it has been suggested that the SPT is not truly reflective of anhedonia in rats (Scheggi et al., 2018). A recent meta-analysis of REMS studies found a small effect size of the procedure on the 10.13039/501100000388SPT, supporting this idea (Stupart et al., 2022). Moreover, sucrose intake may not be an appropriate measure for females, because they show unpredictable increases in sucrose consumption and have an increased tendency to drink more sucrose than males (Dalla et al., 2005). Nonetheless, this would not explain the body weight null results. Multiple studies have reported no effects of REMS, and as discussed in previous sections, other factors may play a role, such as maternal behaviour and increased resilience (Tan et al., 2017). Thus, these discrepancies may arise due to the used anhedonia measures not being sensitive enough to capture the effects of stress, or the REMS procedure not producing the expected outcome.

### 4.7 Neural correlates of habitual control

Our rsFC analysis further implicated several regions known to mediate stimulus-response habit learning. Based on permanent or reversible lesions, the IL is considered to play a critical role in in habitual control (Killcross and Coutureau, 2003; Smith et al., 2012). Furthermore, lesions of the DLS, a striatal node of the putative habit system, facilitate behavioral flexibility by reducing perseverative responding (Graybiel, 2008; Miller et al., 2019; Yin and Knowlton, 2006). In rodents, fronto-striatal reorganization results from chronic stress, which leads to a shift from goal-directed actions to habitual behaviours (Dias-Ferreira et al., 2009; Sharp, 2017). Although there are only few studies in human participants, it has been shown that habitual action selection activates the insular cortex, and activity in the putamen, the human analogue of the rodent DLS, tracks habit development (Eryilmaz et al., 2017; Tricomi et al., 2009). Moreover, the finding that the RL parameter κ is positively correlated with rsFC from the mOFC to the IL, DS and Cg is consistent with evidence showing that inactivation of the mOFC reduces κ (Verharen et al., 2020). It is important to note, however, that the number of animal scans available at PND61/62 was substantially lower than at the second time point (N = 31 vs N = 44), which may have impacted the findings prior to foot-shock stress. Additionally, even though PND61/62 is considered adulthood in rats, the scan at this time point was taken at least 180 days earlier than the beginning of PRL testing at PND241. Some developmental changes may have taken place between those two time points, a limitation to be considered when linking the behavioral measures to rsFC. Therefore, it is plausible that the increase in κ observed in females after adulthood stress was mediated by alterations in the functional coupling strength between the DLS, insular cortex, PFC, and OFC.

### 4.8 Adulthood stress elicits sexually dimorphic effects on functional connectivity

Following a second stressor in adult rats, we observed greater rsFC between the BLA seed and the DLS and AI in male MS rats compared to the control group. This finding provides a possible explanation for the reduction in the learning rate parameter α_non-rew_ seen in controls, aligning with our initial hypothesis that altered learning would be reflected by changes in rsFC between the amygdala and anterior cortical regions. It also provides further support for greater resilience in males exposed to ELS, which were not affected on this parameter. However, no additive effects of stress on connectivity were observed. Lesions of the IL in rats have been shown to cause an impairment in α_non-rew_ and rodent as well as human studies have demonstrated the importance of the DS and BLA in reward learning (Balleine et al., 2007; Jog et al., 1999; Verharen et al., 2020; Wassum and Izquierdo, 2015). ELS in humans reduces rsFC between the amygdala and anterior cingulate cortex, and in MDD there is reduced amygdala-PFC and AI-PFC connectivity, with sex differences also having been reported (Colich et al., 2017; Fan et al., 2014; Kandilarova et al., 2018; Kong et al., 2013). The reduced connectivity to the striatum could be due to hypoactivity in PFC areas resulting from stress, which is also a consequence observed across species (Hayes et al., 2012; Lim et al., 2017; Sailer et al., 2008). The findings presented in our study overlap with human findings, providing evidence for regions affected by REMS that may be of value for future translational research.

In summary, our study explored how RL is altered in rodents exposed to two different stressors. We highlight that female rats are differentially affected by early life and adulthood stress compared to males, as reflected by increased values of the stickiness parameter kappa. Moreover, MS females had lower rsFC from the BLA to PFC regions than their counterparts following adulthood stress, with opposite effects being observed in males. The latter result alongside the reduced α_non-rew_ found in control males, but not MS males, following adulthood stress may be indicative of greater resilience following ELS in males. Collectively, these findings may help to resolve the neurobehavioural basis of sex differences in the etiology of MDD following ELS in humans.

## CRediT authorship contribution statement

**Katharina Zühlsdorff:** Conceptualization, Software, Methodology, Formal analysis, Data curation, Writing – original draft, Writing – review & editing. **Laura López-Cruz:** Conceptualization, Methodology, Investigation, Data curation, Writing – original draft, Writing – review & editing. **Ethan G. Dutcher:** Conceptualization, Methodology, Investigation, Data curation, Writing – original draft, Writing – review & editing. **Jolyon A. Jones:** Methodology, Formal analysis. **Claudia Pama:** Investigation. **Stephen Sawiak:** Methodology, Formal analysis, Investigation, Data curation. **Shahid Khan:** Resources. **Amy L. Milton:** Methodology, Investigation, Writing – review & editing. **Trevor W. Robbins:** Conceptualization, Methodology, Writing – review & editing, Supervision. **Edward T. Bullmore:** Conceptualization, Methodology, Supervision, Resources, Funding acquisition. **Jeffrey W. Dalley:** Conceptualization, Methodology, Writing – review & editing, Supervision, Funding acquisition.

## Declaration of competing interest

The authors declare the following financial interests/personal relationships which may be considered as potential competing interests: **Funding**: This research was funded by a 10.13039/100004330GlaxoSmithKline Varsity Award to JWD, ALM, TWR and ETB (300034212). KZ is funded by the Institute for Neuroscience, University of Cambridge; The Angharad Dodds John Fellowship, Downing College, 10.13039/501100000735University of Cambridge and The 10.13039/100012338Alan Turing Institute, London. EGD acknowledges funding from the 10.13039/501100005370Gates Cambridge Trust. ETB was supported by an 10.13039/501100000272NIHR Senior Investigator Award. **Declaration of conflicting interests:** JWD has received research grants from Boehringer Ingelheim Pharma GmbH and 10.13039/100004330GlaxoSmithKline. TWR is a consultant for Cambridge Cognition and has received research grants from 10.13039/100004330GlaxoSmithKline and Shionogi. ALM has received research grants from 10.13039/501100005612Shionogi. SK is employed by GlaxoSmithKline. ETB is a consultant for Sosei Heptares. The remaining authors declare no conflicts of interest.

## Figures and Tables

**figs1:**
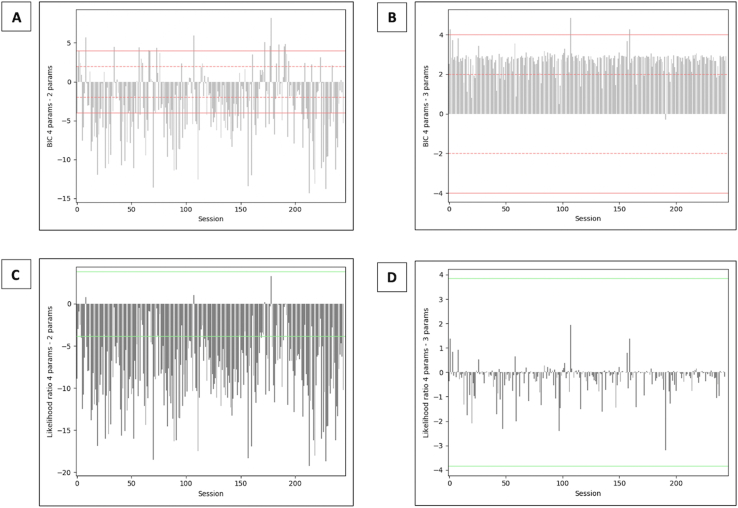


**figs2:**
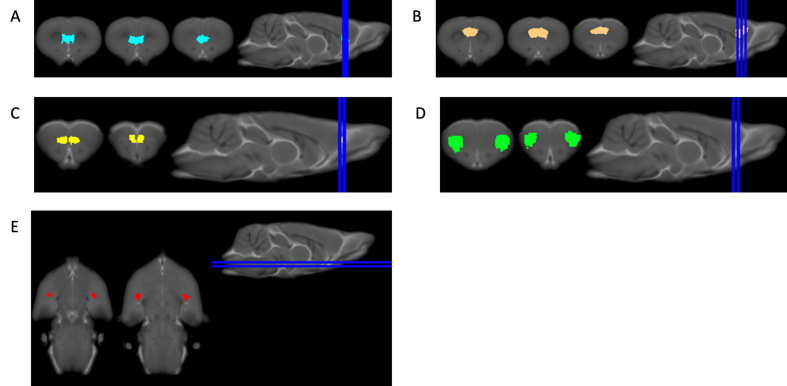


**figs3:**
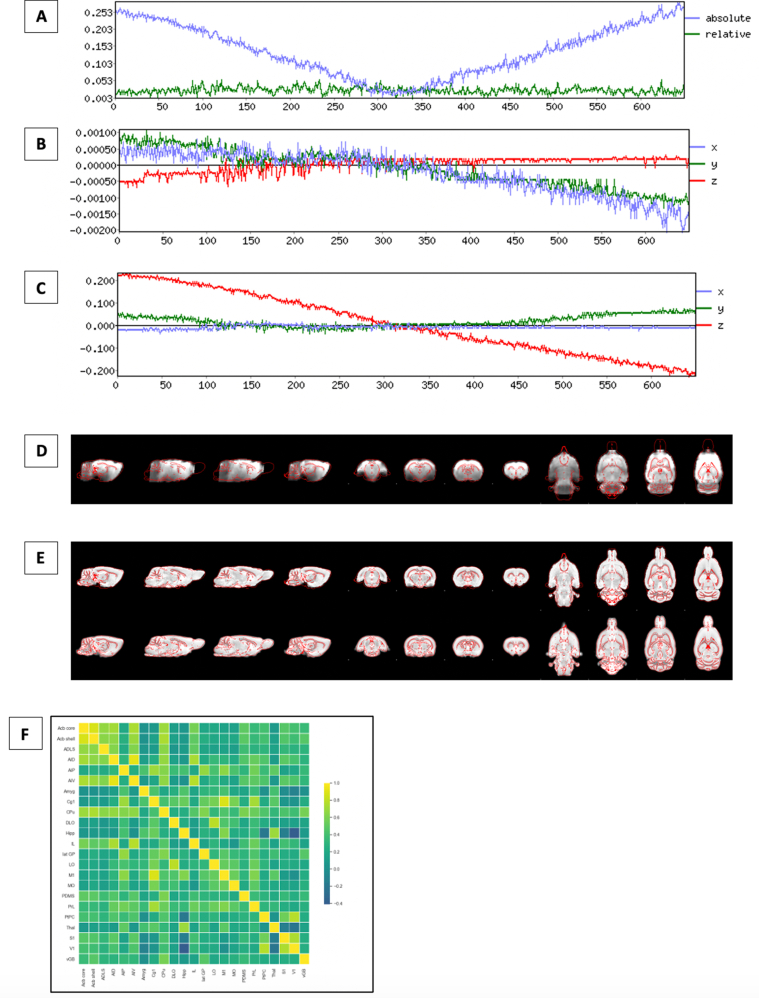


**figs4:**
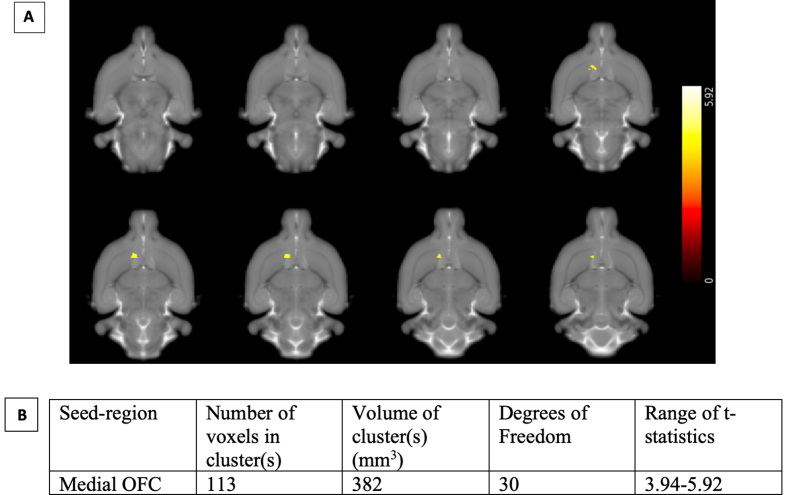


**figs5:**
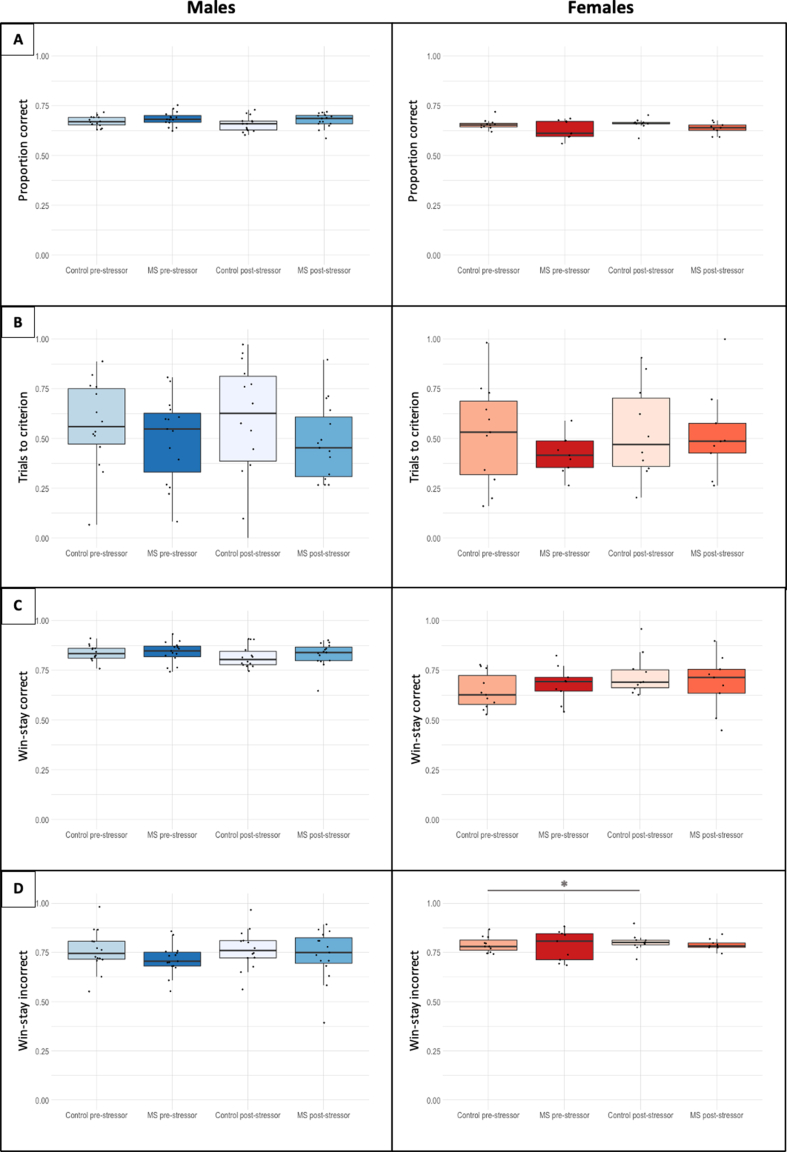


## Data Availability

Behavioral data have been uploaded to Apollo, the link for which can be found in the manuscript. Any other data can be made available on request.
