# Supplementary material for: Sex-dependent effects of early life stress on reinforcement learning and limbic cortico-striatal functional connectivity
Source: Neurobiol Stress. 2022 Dec 5;22:100507. doi: 10.1016/j.ynstr.2022.100507 (PMC9731893; doi:10.1016/j.ynstr.2022.100507)
Supplement: Multimedia component 1 [file mmc1.pdf]

Supplementary materials

Supplementary figure legends

Supplementary Figure 1. Model comparisons of the 4-parameter, 3-parameter and 2-parameter Q-learning models. A) Bayesian Information Criterion (BIC) comparison of the 4-parameter and 2-parameter model, B) BIC comparison of the 4-parameter and 3-parameter model, C) the log likelihood ratio test for the 4- and 2-parameter models, D) the log likelihood ratio test for 4- and 3-parameter models.

The lower the BIC, the more evidence is provided for the model. The dotted lines represent the threshold for a small, but insignificant evidence for one model over the other, and the continuous line represents the threshold for substantial evidence for one model over the other. In the log likelihood ratio test, the significance threshold for comparing models is at 3.842 for p=0.05. Above this threshold, there is a significant difference in data likelihood.
Based on these figures, it can be seen that the 4-parameter model fits the data significantly better than the 2-parameter model, as for the majority of sessions, the BIC for this model is lower than that of the 4-parameter model. The likelihood ratio test confirms this, as the difference between the two models crosses the significance threshold.
The comparison between the 4-parameter and 3-parameter models suggests that both models fit the data equally well. Due to our interest in identifying differences in learning from reward and non-rewarded trials in this study, the 4-parameter model was chosen.

Supplementary Figure 2. Masks of the regions used for the seed-based analysis. A) infralimbic cortex, B) prelimbic cortex, C) medial orbitofrontal cortex, D) lateral orbitofrontal cortex, E) basolateral amygdala.

Supplementary Figure 3. Quality checks conducted on all the functional images to ensure robust pre-processing, including motion correction and registration. A) absolute and relative estimated mean displacement (mm), B) estimated rotations (radians), C) estimated translations (mm), D) example registration of a functional scan to standard space, E) example registration of a structural scan to standard space, F) functional connectivity matrix of a subset of regions at the second time point. The voxel sizes were 1.5 mm. These data were generated using FSL (Smith et al., 2004).

Supplementary Figure 4. A) Areas to which the resting-state connectivity from the medial orbitofrontal cortex (mOFC) is altered due to a sex×maternal separation (MS) interaction. The cluster is mainly located in the ventral striatum (VS), and the connectivity strength between the two regions is higher in control females and MS males than MS females and control males. The color bar on the right-hand side represents the p-values of the respective voxels. B) Table summarizing the main characteristics of the highlighted clusters.

Supplementary Figure 5. Effects of maternal separation (MS) stress on probabilistic reversal learning task in male (blue) and female (orange) rats before and after a second stress during adulthood. The proportion of correct responses and trials to criterion were not significantly affected, neither was win-stay after a correct response. Win-stay after an incorrect response increased post-stressor in control females only ($t(236)=-2.72$, $p=0.0071$). * – $p<0.05$.

Probabilistic reversal learning task

Stage 1 – habituation

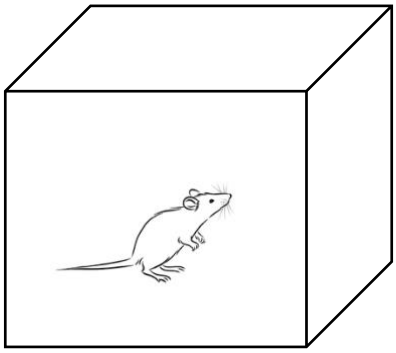

Stage 2 – training to respond to white square stimulus

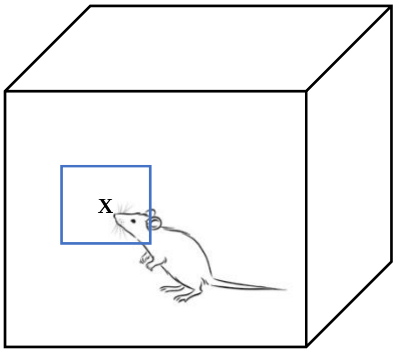

When one of the stimuli was touched, a 0.5 s tone was presented, the pellet receptacle light was turned on and a pellet was delivered. When the pellet was collected, the light turned off and a 5 s inter-trial interval (ITI) was initiated.
After 100 rewards on two consecutive sessions, animals progressed to the next stage.

Stage 3 – any touch outside of the stimulus is punished

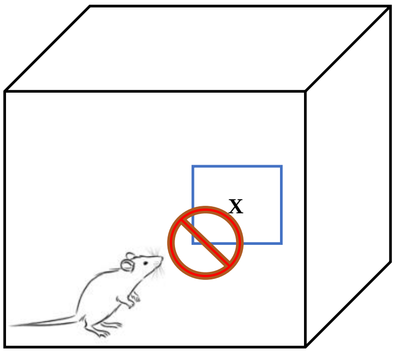

This stage was similar to the previous stage, except that any touch outside of the stimulus resulted in a punishment. The punishment was a 5 s ITI, which had to be initiated by the animal making a head entry after the house light was illuminated for 5 s.
After 100 rewards on two consecutive sessions, animals progressed to the next stage.

Stage 4 – deterministic reversal learning task

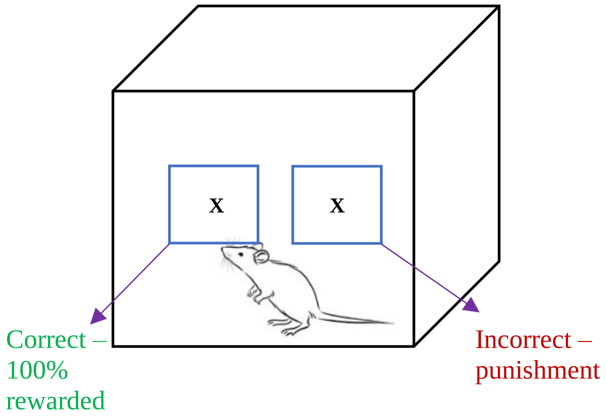

When the correct stimulus was selected 8 times consecutively, the contingencies were reversed. Animals were punished as described in stage 3.
After the contingencies were reversed 4 times in two consecutive sessions, they progressed to the next stage.

Stage 5 – probabilistic reversal learning task

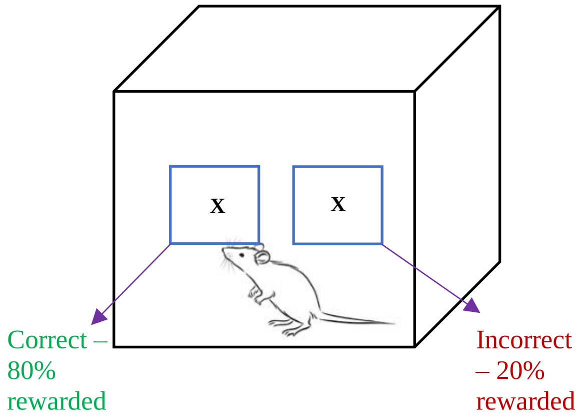

Contingencies were reversed when the animal reached the reversal criterion, which was defined as 8 consecutive responses being correct.

Probabilistic reversal learning task – conventional measure results

Trials to criterion

Factor	DF	F-value	p-value
Sex	1, 44	0.68	0.41
REMS	1, 44	0.79	0.38
Adulthood stress	1, 236	0.086	0.77
Sex×REMS	1, 44	0.02	0.89
Sex×Adulthood stress	1, 236	1.26	0.26
REMS×Adulthood stress	1, 236	0.34	0.56
Sex×REMS×Adulthood stress	1, 236	2.24	0.14

Proportion of correct responses

Factor	DF	F-value	p-value
Sex	1, 44	0.21	0.60
REMS	1, 44	0.10	0.75
Adulthood stress	1, 236	0.045	0.83
Sex×REMS	1, 44	1.67	0.32
Sex×Adulthood stress	1, 236	1.98	0.28
REMS×Adulthood stress	1, 236	0.61	0.44
Sex×REMS×Adulthood stress	1, 236	0.71	0.40

Win-stay correct

Factor	DF	F-value	p-value
Sex	1, 44	8.85	0.0048
REMS	1, 44	0.002	0.97
Adulthood stress	1, 236	0.070	0.79
Sex×REMS	1, 44	0.324	0.57
Sex×Adulthood stress	1, 236	2.37	0.13
REMS×Adulthood stress	1, 236	0.067	0.80
Sex×REMS×Adulthood stress	1, 236	0.25	0.62

Win-stay incorrect

Factor	DF	F-value	p-value
Sex	1, 44	4.11	0.049
REMS	1, 44	0.66	0.42
Adulthood stress	1, 236	6.04	0.015
Sex×REMS	1, 44	0.97	0.33
Sex×Adulthood stress	1, 236	0.55	0.46
REMS×Adulthood stress	1, 236	0.33	0.57
Sex×REMS×Adulthood stress	1, 236	3.00	0.084

Lose-shift correct

Factor	DF	F-value	p-value
Sex	1, 44	3.51	0.068
REMS	1, 44	0.41	0.52
Adulthood stress	1, 236	2.34	0.13
Sex×REMS	1, 44	0.33	0.57
Sex×Adulthood stress	1, 236	0.60	0.44
REMS×Adulthood stress	1, 236	0.72	0.40
Sex×REMS×Adulthood stress	1, 236	4.16	0.043

Lose-shift incorrect

Factor	DF	F-value	p-value
Sex	1, 44	3.86	0.056
REMS	1, 44	0.49	0.49
Adulthood stress	1, 236	0.22	0.64
Sex×REMS	1, 44	4.33	0.043
Sex×Adulthood stress	1, 236	0.058	0.81
REMS×Adulthood stress	1, 236	0.10	0.75
Sex×REMS×Adulthood stress	1, 236	7.49	0.0067

Perseverative responses

Factor	DF	F-value	p-value
Sex	1, 44	0.0013	0.97
REMS	1, 44	0.069	0.79
Adulthood stress	1, 236	2.01	0.16
Sex×REMS	1, 44	7.19	0.010
Sex×Adulthood stress	1, 236	8.29	0.0043
REMS×Adulthood stress	1, 236	3.14	0.078
Sex×REMS×Adulthood stress	1, 236	0.79	0.37

Q-learning model results

Alpha_rew: learning rate from rewarded trials

Factor	DF	F-value	p-value
Sex	1, 44	0.23	0.63
REMS	1, 44	1.79	0.19
Adulthood stress	1, 236	1.23	0.29
Sex×REMS	1, 44	0.18	0.67
Sex×Adulthood stress	1, 236	0.09	0.76
REMS×Adulthood stress	1, 236	1.96	0.16
Sex×REMS×Adulthood stress	1, 236	3.46	0.064

Alpha_non-rew: learning rate from non-rewarded trials

Factor	DF	F-value	p-value
Sex	1, 44	0.64	0.43
REMS	1, 44	2.35	0.13
Adulthood stress	1, 236	3.66	0.057
Sex×REMS	1, 44	0.67	0.42
Sex×Adulthood stress	1, 236	0.23	0.64
REMS×Adulthood stress	1, 236	4.14	0.043
Sex×REMS×Adulthood stress	1, 236	0.15	0.69

Beta: exploitation vs exploration parameter

Factor	DF	F-value	p-value
Sex	1, 44	1.45	0.29
REMS	1, 44	0.24	0.63
Adulthood stress	1, 236	1.55	0.21
Sex×REMS	1, 44	0.027	0.87
Sex×Adulthood stress	1, 236	1.02	0.31
REMS×Adulthood stress	1, 236	0.006	0.94
Sex×REMS×Adulthood stress	1, 236	0.19	0.67

Kappa: autocorrelation/‘stickiness’ parameter

Factor	DF	F-value	p-value
Sex	1, 44	1.94	0.17
REMS	1, 44	0.60	0.44
Adulthood stress	1, 236	0.28	0.60
Sex×REMS	1, 44	0.24	0.63
Sex×Adulthood stress	1, 236	0.00030	0.99
REMS×Adulthood stress	1, 236	0.14	0.71
Sex×REMS×Adulthood stress	1, 236	8.20	0.0046
